# Compositional magnetic resonance imaging in the evaluation of the intervertebral disc: Axial vs sagittal T_2_ mapping

**DOI:** 10.1002/jor.24691

**Published:** 2020-06-05

**Authors:** Marcus Raudner, Markus M. Schreiner, Michael Weber, Vladimir Juras, David Stelzeneder, Reinhard Windhager, Siegfried Trattnig

**Affiliations:** ^1^ Department of Biomedical Imaging and Image‐guided Therapy Medical University of Vienna Vienna Austria; ^2^ Department of Biomedical Imaging and Image‐guided Therapy, Christian Doppler Laboratory for Clinical Molecular Magnetic Resonance Imaging (MOLIMA), High Field MR Center Medical University of Vienna Vienna Austria; ^3^ Department of Orthopedics and Trauma Surgery Medical University of Vienna Vienna Austria; ^4^ Department of Orthopedics and Trauma Surgery Hanusch Hospital Vienna Austria

**Keywords:** degenerative disc disease, intervertebral disc, low back pain, magnetic resonance imaging, T_2_ mapping

## Abstract

The aim of this study was to assess T_2_ values of the lumbar intervertebral discs in the axial and sagittal plane views and assess their respective interobserver reliability. The lumbar intervertebral discs of 23 symptomatic patients (11 female; 12 male; mean age, 44.1 ± 10.6; range, 24‐64 years) were examined at 3T. Region‐of‐interest (ROI) analysis was performed on axial and sagittal T_2_ maps by two independent observers. Intraclass correlation coefficient (ICC) was assessed for every ROI. The interobserver agreement was excellent for the nucleus pulposus (NP) in the sagittal (0.951; 95% confidence interval [CI], 0.926‐0.968) and axial (0.921; 95% CI, 0.845‐0.955) planes. The posterior 20% region showed a higher ICC in the axial vs the sagittal assessment (0.845; 95% CI, 0.704‐0.911 vs 0.819; 95% CI, 0.744‐0.873). The same was true for the posterior 10%, with the axial ROI showing a higher ICC (0.923; 95% CI, 0.865‐0.953 vs 0.628; 95% CI, 0.495‐0.732). The intraobserver agreement was excellent for every ROI except the sagittal 10% region, which showed good performance (0.869; 95% CI, 0.813‐0.909). The sagittal nucleus pulposus was the best‐performing ROI with regard to intra‐ and interobserver agreement in the T_2_ assessment of the lumbar intervertebral disc. However, the axial NP showed more stable agreements overall and across the value range. In addition, the annular analysis showed better inter‐ and intraobserver agreement in the axial plane view. Clinical significance: Based on the presented analysis, we highly recommend that further studies use axial T_2_ mapping due to the higher intra‐ and interreader agreement.

## Graphical abstract

In this study, T_2_ maps of 23 symptomatic low back pain patients measured at 3T were assessed in axial and sagittal plane view by two independent observers. The sagittal nucleus pulposus was the best‐performing region‐of‐interest with regard to intra‐ and interobserver agreement in the T_2_ assessment of the lumbar intervertebral disc. However, the axial NP showed more stable agreements overall and across the value range. In addition, the annular regions showed better inter‐ and intraobserver agreement in the axial plane view.

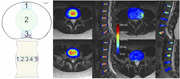

**Abbreviations**
AF: annulus fibrosus
IVD: intervertebral disc
LBP: low back pain
MRI: magnetic resonance imaging
NP: nucleus pulposus
ROI: region‐of‐interest
T_2_w: T_2_‐weighted

## 1 INTRODUCTION

As the leading cause for both years lived with disability (YLDs) and disability‐adjusted life years (DALYs) according to the Global Burden of Disease Studies of 2016 and 2013, low back pain (LBP) must be considered one of the most important socioeconomic factors of our current generation.^1^, ^2^

Back pain is typically caused by pathological changes, including degenerative disc disease (DDD), disc herniation, spondylolysis, spondylolisthesis, vertebral fractures, and scoliosis,^3^ and, with smoking and obesity the general risk factors for LBP,^4^ it is hardly surprising that approximately two‐thirds of adult patients present with clinically relevant symptoms.^5^ In addition, during the first 12 months after onset, every fourth patient relapses, increasing the risk for long‐term disability.^6^

The most prominent of the aforementioned causes for LBP is degenerative disc disease (DDD), which is defined as progressive and irreversible structural failure of the disc's compartments that results in biomechanical dysfunction.^7^ DDD is thought to account for up to 45% of LBP, with resulting disc herniations, annular fissures, and osteochondrosis acting as catalysts for further degenerative changes in the spine, which subsequently results in chronic pain.^8^ With the consecutive inflammatory upregulation, both neovascularization and in‐growth of sinuvertebral nerve endings can be shown in highly degenerated intervertebral disc (IVDs). These sinuvertebral nerve endings are thought to be of the visceral type and cause the agonizing character of LBP.^8^, ^9^

While acute LBP is not an indication for routine magnetic resonance imaging (MRI), for neurological deficits, suspected disc herniation, persistence over three months, or if other diseases must be ruled out, MRI is the established method of choice.^10^, ^11^, ^12^

In MRI, disc degeneration is usually assessed using the Pfirrmann score, grading the signal intensity of the nucleus pulposus (NP) and its distinction from the annulus fibrosus (AF) on sagittal T_2_‐weighted (T_2_w) images.^13^

However, there are efforts to establish quantitative MRI in the clinical routine. As shown in previous studies, T_2_ relaxation time measurement can be used to assess the ultrastructural integrity of the IVD compartments. The surveyed T_2_ values correlate with the water‐storing capabilities of the NP and the collagen fiber density of the surrounding AF. This is why prior studies have already stated that deploying T_2_ mapping of the IVDs in the clinical routine may complement assessment.^14^, ^15^, ^16^, ^17^

In healthy IVDs, high T_2_ values are expected in the NP due to its water‐storing capabilities, and low T_2_ values are considered physiological in the annular regions, indicating an intact and dense collagen network.^18^, ^19^

Using a conventional multi‐echo spin echo Carr‐Purcell‐Meiboom‐Gill sequence, the relaxation time is measured using a conventional radiofrequency (RF) pulse of 90°, with subsequent refocusing pulses of 180° to minimize T_2_* effects from the dephasing spins and maintain the T_2_‐weighting.^20^ These refocusing pulses are repeated in equidistant steps as often as needed to cover the whole spectrum of typical T_2_ values given the measured area of interest. T_2_ mapping is able to reveal very early stages of disc degeneration that are invisible or highly underappreciated on conventional morphological sequences.^21^ T_2_ mapping is sensitive enough to detect differences due to diurnal changes and can quantitatively reflect the changing water content after about 30 minutes of short‐term unloading.^22^, ^23^

T_2_ mapping also correlates with histology, and temporary changes in water distribution after high load can be shown alongside short‐term regeneration.^22^, ^24^ Overall, T_2_ mapping can be considered a desirable imaging biomarker.^15^, ^25^

However, T_2_* mapping can be used with shorter acquisition times and the potential of three‐dimensional isotropic sequences.^26^ T_2_* is always shorter than T_2_ and reflects the real‐life T_2_, as dephasing spins are not corrected for by 180° refocusing pulses. However, T_2_* is highly susceptible to magnetic field inhomogeneities, which is why it is often used in the clincal routine to depict hemorrhages or calcifications.^27^ T_2_* can be of use in the lumbar IVD, but has been shown to be insensitive to diurnal changes or effects from short‐term unloading.^28^

Whereas, in clinical practice, morphological assessment is typically performed in both the axial and sagittal planes, thus far, there have been no studies that have evaluated either interobserver variability, or the ability to discriminate between herniated or degenerated discs on axial and sagittal compositional MR, in general. More specifically, T_2_ relaxometry has not been studied with regard to this issue. Thus, the purpose of this study was to compare the inter‐ and intraobserver agreement of sagittal and axial T_2_ mapping of the lumbar IVD through direct comparison in the same patient cohort.

## 2 METHODS

### 2.1 Patients and study design

This study was a case‐control (level of evidence: III) MRI study to investigate sagittal and axial T_2_ mapping in patients with lower back pain. Upon the approval of the ethical review board, 25 symptomatic patients were enrolled directly from the orthopedics outpatient clinic.

Inclusion criteria were LBP (single or recurrent episodes) and age between 15 and 65 years. Exclusion criteria were neurological deficits of the lower limbs, radicular pain, a body mass index greater than 30, previous surgical interventions of the lumbar IVDs, scoliosis with a Cobb‐angle exceeding 15°, recently diagnosed disc herniation (<12 months), and contraindications for MRI.

Of the 25 enrolled patients, one patient had to stop the examination due to pain in the prolonged supine position and another was discarded due to motion artifacts. This resulted in 23 patients evaluated in the presented study (11 female; 12 male; mean age, 44.1 ± 10.6 years; range, 24‐64). Written, informed consent was obtained from all enrolled patients.

### 2.2 MRI

All MR examinations were performed using a 3T Siemens MAGNETOM Trio (Siemens Healthineers, Erlangen, Germany) with a gradient strength of 40 mT/m and a dedicated eight‐channel spine coil (both Siemens Healthineers, Erlangen, Germany). Morphological assessment was performed using sagittal, axial, and coronal T_2_w, as well as sagittal T_1_w images.

T_2_ relaxation time measurements were conducted in the axial and sagittal plane views, with a time to repetition of 1200 millisecond, an echo train length of 6, with time to echos 13.8, 27.6, 41.4, 55.2, 69.0, and 82.8 millisecond. All sequence parameters are presented in Table 1.

**Table 1:** MR sequence parameters; no interslice gap is given for the axial T_2_ mapping sequence, as only one slice per disc was acquired

	T_1_w TSE sag	T_2_w TSE sag	T_2_w TSE axial	T_2_w TSE cor	T_2_w TSE STIR sag	T_2_ map sag	T_2_ map axial
Time to repetition, ms	900	4400	5080	4500	3500	1200	1200
Time to echo, ms	8.3	105	94	105	35	13.8; 27.6; 41.4; 55.2; 69.0; 82.8	13.8; 27.6; 41.4; 55.2; 69.0; 82.8
Field of view, mm	300 × 300	280 × 280	210 × 210	280 × 280	300 × 300	220 × 220	220 × 220
Matrix size	320 × 320	320 × 320	384 × 384	320 × 320	320 × 320	256 × 256	256 × 256
Voxel size, mm	0.9 × 0.9	0.9 × 0.9	0.7 × 0.7	0.9 × 0.9	0.9 × 0.9	0.9 × 0.9	0.9 × 0.9
Slice thickness, mm	3	3	3	3	4.0	5	3
Interslice gap, mm	0.3	0.3	0.3	0.3	0.4	1	n/a
Number of slices	15	15	8 × 5	15	15	10	5
Scan time, min:s	03:23	01:34	06:16	01:36	3:25	07:41	07:41

Abbreviation: MR, magnetic resonance.

T_2_ maps were calculated on‐site using MapIt (Siemens Healthineers, Erlangen, Germany). All patients had a lower leg support (15 cm maximum height) placed accordingly during the examination.

### 2.3 Image analysis

A senior radiologist with over two decades of experience in musculoskeletal MRI issued radiological reports, including degeneration grading and classification of disc pathologies based on Fardon et al.^29^ A radiology resident and an orthopedic resident, both with over 5 years of experience with musculoskeletal MRI studies, independently evaluated the axial and sagittal T_2_ maps on a syngo.via LEONARDO console (Siemens Healthcare, Erlangen, Germany). Patients were assessed in random order to minimize recall bias and both readers were blinded to all patient details.

The disc was subdivided based on strict geometrical rules, with the outer 40% of the disc representing the AF (the anterior and posterior 20%, respectively) and the inner 60% representing the NP.^30^ Excellent inter‐ and intraobserver reliability have already been demonstrated for this assumption.^14^

**Figure 1:**
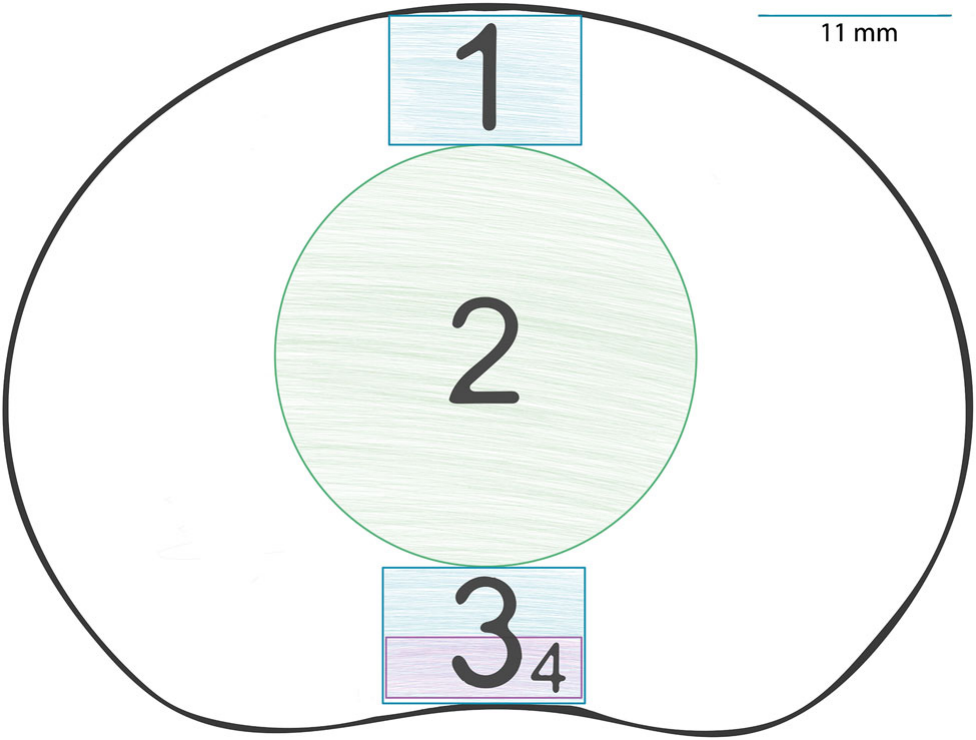
Axial disc evaluation and region‐of‐interest (ROI) placement. 1: Anterior annulus fibrosus – discarded in this evaluation, but used as a placeholder for reproducibility; 2: nucleus pulposus; 3: posterior annular 20% region; 4: posterior annular 10% region. ROIs 1, 3, and 4 were drawn with a width of 11 mm to match the sagittal assessment of the annular region [Color figure can be viewed at wileyonlinelibrary.com]

The posterior annulus fibrosus (PAF) was sampled with a region‐of‐interest (ROI) size of 20% (PAF20) and subdivided again with a dedicated ROI for the most posterior 10% (PAF10) of the length of the disc in the sagittal plane based on the work of Messner et al.^31^ This emphasizes the idea of advancing degradation and tearing of annular regions starting from the transitional inner zone of the disc and progressively migrating to the outer areas. An illustration of the sagittal segmentation is given in Figure 1.

**Figure 2:**
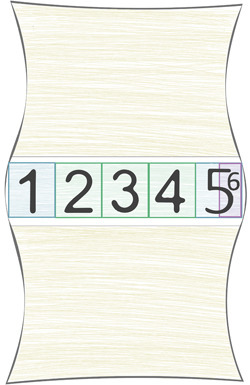
Sagittal disc evaluation and region‐of‐interest (ROI) placement. 1: Anterior annulus fibrosus (AF) – discarded in this evaluation, but used as a placeholder for reproducibility; 2‐4: nucleus pulposus (NP) – assessed separately to avoid artifacts or other sources of false values, and aggregated later; 5: posterior annular 20% region, and 6: posterior annular 10% region. Since two central sagittal slices were assessed, the thickness of the evaluated disc area was 5 mm + 5 mm + 1 mm gap, resulting in the central 11 mm of the examined discs being used for the quantitative evaluation [Color figure can be viewed at wileyonlinelibrary.com]

On axial T_2_ maps, the nucleus was assessed by placing one circular ROI with a diameter of 60% of the anterior to posterior diameter of the center of the disc. This was the only systematic offset intended to depict possible changes in the values assessed by including more voxels on both lateral sides of the disc's NP. The sagittal assessment was done only on the two most central slices to avoid partial volume effects and errors, in general, especially on more degenerated discs. The axial segmentation is schematically depicted in Figure 2.

All segmentations were conducted by two blinded observers, a radiology resident (reader 1) and an orthopedics resident (reader 2), both with 4 years of dedicated experience in musculoskeletal MRI studies. Reader 1 repeated the evaluation 3 weeks after the first assessment.

### 2.4 Statistical evaluation

All statistical analyses were performed by a biomedical statistician using IBM SPSS for Windows version 25 (IBM, Chicago, IL). Categorical data are presented as absolute frequencies and percentages, and metric data are described using mean ± SD. Intraclass correlation coefficients (ICCs) were interpreted according to Koo and Li.^32^ An ICC of less than 0.50 indicated poor agreement, an ICC of 0.50‐0.75 moderate agreement, and ICC of 0.75‐0.90 good agreement, and an ICC greater than 0.90 excellent agreement. ICCs are given with 95% confidence intervals (CIs). To account for multiple measurements per patient, a hierarchical linear model was used to evaluate differences between readers and axial vs sagittal assessment. A *P*‐value of less than or equal to .05 was considered to indicate statistically significant results.

## 3 RESULTS

### 3.1 NP assessment

Excellent interobserver agreement could be demonstrated for the sagittal (0.951; 95% CI, 0.926‐0.968) and axial (0.921; 95% CI, 0.845‐0.955) evaluation of the NP. The same was true for the intraobserver agreement for sagittal (0.973; 95% CI, 0.961‐0.982) and axial (0.966; 95% CI, 0.951‐0.976) assessment. For an overview of all inter‐ and intraobserver agreements, see Table 2.

**Table 2:** Mean values per ROI for both readers ± SD with the calculation of two‐way mixed absolute agreement ICC with 95% CI

	Reader 1	Reader 2	Reader 1 2nd read	Interrater reliability (ICC)	Intrarater reliability (ICC) reader 1
Sagittal NP	103.7 ± 37.4	107.0 ± 41.9	104.0 ± 35.7	0.951 (0.926‐0.968)	0.973 (0.961‐0.982)
Axial NP	95.0 ± 25.2	99.7 ± 30.2	95.3 ± 25.9	0.921 (0.845‐0.955)	0.966 (0.951‐0.976)
Sag posterior 20%	59.3 ± 14.8	60.6 ± 13.5	58.4 ± 16.0	0.819 (0.744‐0.873)	0.920 (0.884‐0.945)
Axial posterior 20%	53.0 ± 10.5	55.9 ± 11.6	53.2 ± 11.3	0.845 (0.704‐0.911)	0.924 (0.892‐0.947)
Sag posterior 10%	46.4 ± 8.0	46.4 ± 8.7	45.9 ± 9.5	0.628 (0.495‐0.732)	0.869 (0.813‐0.909)
Axial posterior 10%	46.6 ± 11.3	48.4 ± 11.9	46.1 ± 11.1	0.923 (0.865‐0.953)	0.936 (0.909‐0.956)

Abbreviations: CI, confidence interval; ICC, Intraclass correlation coefficient; NP, nucleus pulposus; ROI, region‐of‐interest.

The hierarchic linear model showed significant differences between both readers, with reader 1 achieving higher T_2_ values than reader 2 (*P* < .001). Also, there was a significant difference between assessments (*P* = .002) with higher T_2_ values in the sagittal assessment for both readers. The differences in the assessment did not significantly differ between raters (*P* = .303). The intrarater analysis of the hierarchical model showed that there was no significant difference between the first and second read (*P* = .506), but a significantly higher T_2_ in the sagittal assessment (*P* < .001).

### 3.2 AF assessment: Posterior 20%

The sagittal evaluation showed good interobserver agreement (0.819; 95% CI, 0.744‐0.873) in the assessment of the posterior 20% region. The same was true for the axial assessment (0.845; 95% CI, 0.704‐0.911) assessment of the posterior 20% region. The intraobserver agreement was excellent in the sagittal (0.920; 95% CI, 0.884‐0.945) and axial (0.924; 95% CI, 0.892‐0.947) assessment. The hierarchic linear model showed significant differences between both readers (*P* < .001), with reader 1 achieving lower T_2_ values than reader 2.

Also, there was a significant difference between assessments with higher T_2_ values in the sagittal assessment for both readers (*P* < .001). The differences in the assessment did not significantly differ between raters (*P* = .091). The intrarater analysis of the hierarchical model showed that there was no significant difference between the first and second read (*P* = .384), but a significantly higher T_2_ in the sagittal assessment (*P* = .001).

### 3.3 AF assessment: Posterior 10%

The sagittal assessment showed only moderate interobserver agreement in the sagittal assessment of the posterior 10% region (0.628; 95% CI, 0.495‐0.732). However, excellent agreement could be demonstrated for the axial (0.923; 95% CI, 0.865‐0.953) assessment. The intraobserver agreement was excellent for the axial (0.936; 95% CI, 0.909‐0.956) assessment and good for the sagittal (0.869; 95% CI, 0.813‐0.909) assessment.

The hierarchic linear model showed significant differences between both readers (*P* < .025), with reader 1 achieving lower T_2_ values than reader 2. Also, there was a significant difference between assessments with higher T_2_ values in the sagittal assessment for both readers (*P* < .001). The differences in the assessment did not significantly differ between raters (*P* = .376). The intrarater analysis of the hierarchical model showed that there was no significant difference between the first and second read (*P* = .052) or between sagittal and axial assessment (*P* = .891).

## 4 DISCUSSION

In this study, we investigated T_2_ values of the lumbar IVDs in the sagittal and axial planes to evaluate the ICC for the different ROI placements. The main finding of this study is that the axial analysis showed a trend toward better interobserver and intraobserver agreement in both posterior annular regions, with excellent agreement for the NP in both assessments.

The highest inter‐ and intrarater agreements were shown for the sagittal nucleus pulposus assessment. The lowest inter‐ and intrarater agreements were found in the sagittal assessment of the posterior 10% region, as seen in Table 2. Most study groups use sagittal T_2_ mapping in the assessment of lumbar IVDs.^14^, ^31^, ^33^

To our knowledge, this is the first study to provide an overview of subsequent sagittal and axial T_2_ mapping in an identical patient cohort. The presented data illustrate that both sagittal and axial assessment can be used for the NP, yielding excellent inter‐ and intrarater agreement. However, both annular regions showed better inter‐ and intrarater agreement in the axial assessment.

**Figure 3:**
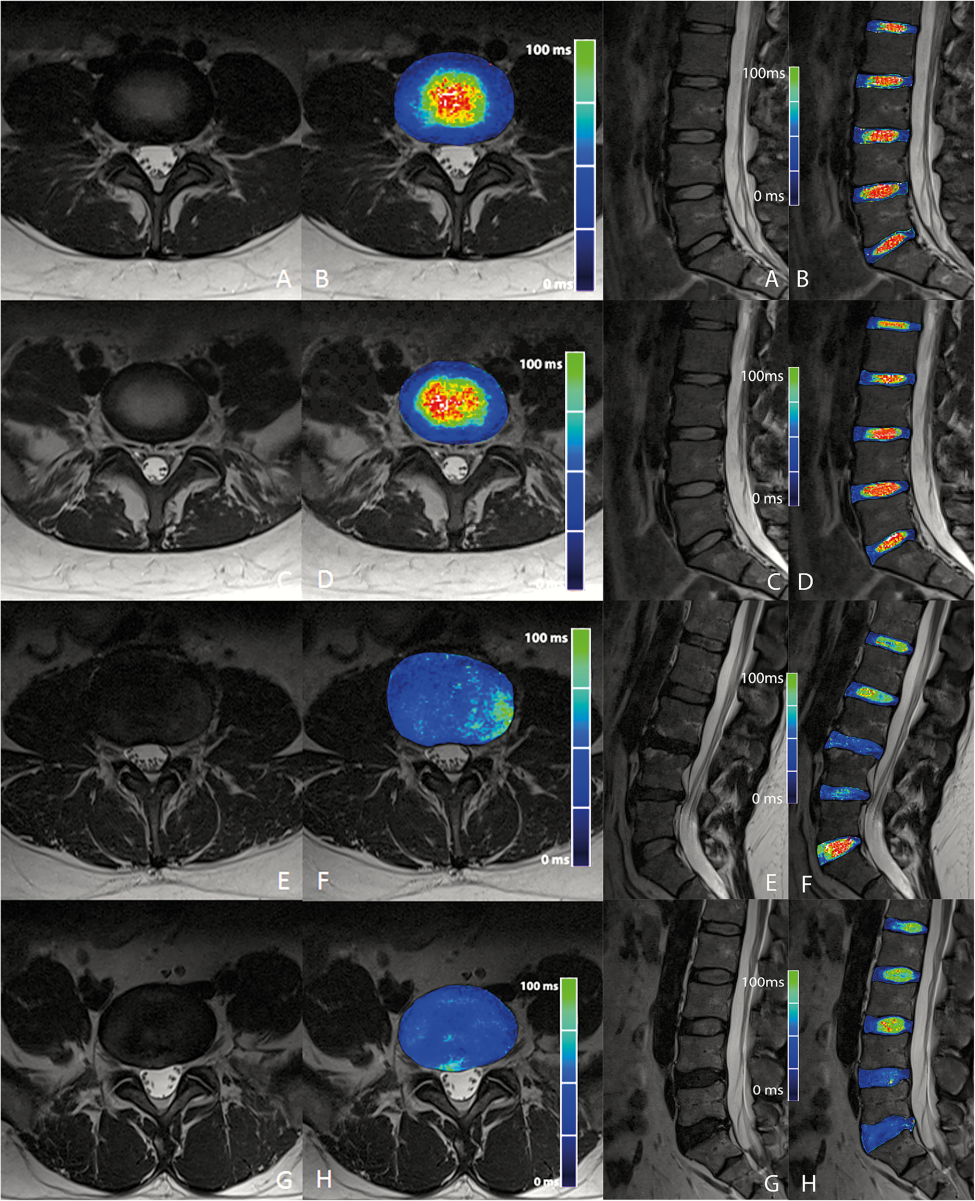
Comparison of morphological T_2_w images and color‐coded T_2_ map overlay in the axial and sagittal planes: A and B, normal L4/L5 disc (axial) and sagittal overview; C and D, axial: normal L5/S1 disc and sagittal overview; E and F, axial: severely degenerated L3/L4 disc with lateral protrusion, which is not seen on the sagittal overview and could be missed depending on the number of slices; G and H, severely degenerated L5/S1 disc with posterior protrusion and annular tear, better depicted in the axial plane view [Color figure can be viewed at wileyonlinelibrary.com]

Therefore, axial T_2_ mapping might be beneficial for a thorough biochemical analysis of the IVDs, especially when focusing on annular pathologies, such as high‐intensity zones. Also, early degeneration stages and medio‐lateral and lateral herniation or annular fissure could be better visualized in the axial vs the sagittal plane view.^34^ Example images are given in Figure 3.

The NP T_2_ values are known to decrease with disc degeneration while the T_2_ increases in the annular regions. Previous studies have already shown that T_2_ mapping can differentiate between Pfirrmann scoring in interverbal discs.^14^, ^15^, ^25^, ^35^, ^36^

Even tough annular fissures do not definitively have a significant impact as an isolated finding, as clinical patients who are referred for an MR due to pain often show a loss of physiological integrity of the effected discs. Consequently, imaging this on a biochemical level using T_2_ mapping can help in the clinical routine.^31^, ^37^, ^38^

As human trials of T_2_ mapping of the IVDs are almost exclusively conducted in the sagittal plane view, data regarding axial assessment of the disc using biochemical MRI are scarce.^39^, ^40^ However, our results are promising for axial T_2_ mapping of the IVD, with increased intra‐ and interrater reliability for the annular regions in the axial vs sagittal disc assessment in the same cohort. This could lead to better repeatability and higher comparability for future studies or multicenter trial settings. Our data suggest that axial T_2_ mapping is a feasible and reproducible method with which to assess the biochemical state of the IVD and to evaluate degenerative disc disease.

The limitations of our study include the fact that only a single axial slab was used per disc, as opposed to the sagittal T_2_ map evaluation, which was conducted on two sagittal slices, making the axial T_2_ value assessment dependent on the radiographers' accuracy. This is the case especially in discs with morphological abnormalities or collapsed disc spaces, which, however, was not a confounder in our data. Another limitation is that no healthy controls were examined in this study. However, patients regularly show one or more discs with no severe degeneration, therefore, providing enough potential normal T_2_ values to cover the spectrum.

Also, the NP was very differently assessed in the axial vs the sagittal evaluation. This was intentional, however, as one of the main goals was to see whether the axial assessment with its bigger NP ROI provides any additional value, as sagittal studies typically evaluate only the most central slices to minimize potential inaccuracies caused by partial volume effects in the more lateral regions in the sagittal plane view. It has to be noted, in this regard, that the highest differences between axial and sagittal assessments were seen in the posterior 10% region. Even if this ROI was very strictly defined and assessed as comparable as possible geometrically, both intra‐ and interrater agreement favored the transversal approach. This is likely caused by the fact that the axial ROIs were bigger, voxel‐wise, as the in‐plane resolution increased ROI size and voxel count even if the geometrical placement was identical to that in the sagittal plane.

Overall, we conclude that axial T_2_ mapping offers superior inter‐ and intrarater correlation coefficients. Sagittal T_2_ mapping remains the more established method, with slightly better ICC in our analysis. However, the annular regions showed substantially better inter‐ and intrareader reliability in the axial assessment. Therefore, further studies involving axial T_2_ mapping are warranted.

## CONFLICTS OF INTEREST

All the authors declare that there are no conflicts of interest.

## AUTHOR CONTRIBUTIONS

MR, MS, MW, and ST provided substantial contributions to research design, or the acquisition, analysis, or interpretation of data; VJ assisted in drafting the paper or revising it critically and approval of the submitted and final versions; DS gave substantial contributions to research design, or the acquisition, analysis, or interpretation of data and approval of the submitted and final versions; and RW helped in the approval of the submitted and final versions. All authors have read and accepted the following manuscript in its final form and state their respective work is adequately represented in it by the chosen author succession.
